# Chemico-calorimetric analysis of amorphous granules manufactured via continuous granulation process

**DOI:** 10.1007/s13346-018-0519-3

**Published:** 2018-04-24

**Authors:** Mridul Majumder, Saeid Rajabnezhad, Ali Nokhodchi, Mohammed Maniruzzaman

**Affiliations:** 1 M2M Pharmaceuticals Ltd., The Gateway Building, 1 Collegiate Square, Thames Valley Science Park (TVSP), Reading, RG2 9LH United Kingdom; 2 Department of Pharmacy/Chemistry, School of Life Sciences, University of Sussex, Falmer, Brighton, BN1 9QJ UK; 3 Drug Applied research Center and Faculty of Pharmacy, Tabriz Medical Sciences University, Tabriz, Iran

**Keywords:** Granules, Twin screw granulations, Amorphous, Solution calorimetry, DVS

## Abstract

The current study explores the first case of the implementation of solution calorimetry (SolCal) in order to determine the amorphous content of crystalline benzoyl-methoxy-methylindol-acetic acid (BMA)—a model poorly soluble drug, in the amorphous granules prepared via single-step continuous twin-screw dry granulations (TSG). Amorphous magnesium aluminometasilicate (Neusilin®) (US2) was used as a novel inorganic carrier via a TwinLab 10 mm twin-screw extruder. The BMA/US2 blends were processed at 180 °C and varying drug: carrier ratios of 1:4, 1:2.5 and 1:1 (*w*/*w*). Physico-chemical characterisation conducted via SEM, DSC and XRPD showed amorphous state of the drug in all granulated formulations. Reverse optical microscopy revealed a meso-porous structure of US2 in which the drug particles are adsorbed and/or entrapped within the porous network of the carrier. This phenomenon can be the underlying reason for the increase of the amorphous content in the extruded granules. Solution calorimetry (SolCal) study revealed amorphous content of the drug in all formulations quite precisely, whereas the dynamic vapour sorption (DVS) analysis complemented the results from SolCal. Furthermore, an attempt has been made for the first time to interrelate the findings from the SolCal to that of the release of the drug from the amorphous granules. It can be concluded that SolCal can be used as a novel technique to precisely quantify and interrelate the amorphous content to its physico-chemical performances such as drug release from the granulated formulations processed via TSG.

## Introduction

It has been reported that about 40% of the new lead compounds found in the drug discovery pipeline are becoming unsuitable due to its inherent poor water solubility or insolubility issue [1, 2]. Thus, solubility enhancement of these poorly water-soluble drugs remains a fundamental challenge for the pharmaceutical scientists across the industry. Despite numerous studies on advanced manufacturing processes, there seems to be no versatile technique currently available that can tackle this growing solubility issue for all drugs. There have been various methods and approaches reported to increase the solubility thus the dissolution rates of the poorly water soluble drugs such as hot spin mixing [3], spray drying [4], co-evaporation or co-precipitation [5], freeze-drying [6], supercritical fluid processing (SFP) [7], hot-melt extrusion (HME) and recently twin-screw granulations (TSG) using HME techniques [8, 9]. The ultimate aim of the majority if not all of these reported techniques is to obtain an amorphous solid of the drug candidate due to the fact that amorphous forms of crystalline counterpart have always been appeared as an evolving phenomenon in pharmaceutical sciences [10]. Amorphous form of a drug generally appears in more highly energetic state compared to the crystalline form which often proves beneficial in increasing solubility and thus bioavailability [11, 12].

Recently, twin screw granulations (TSG) have attracted considerable attention especially in the area of pharmaceutical formulation development. Most of the reported studies in the past decades or so have mainly described the production of semi-crystalline granules or agglomerates via two major extrusion granulation techniques: (i) wet, and (ii) hot melt or dry granulation [11–14]. The latter being more suitable for the continuous operation as it eliminates intermediate steps by producing free-flowing ready-to-use granules [12]. These granules then can be incorporated into the final dosage forms such as tablets. However, a suitable formulation has to be developed in order to process any challenging molecules such as heat-sensitive excipients like vitamins or proteins. Recently, a new developed concept of one step continuous solvent-free extrusion (a process where hot dry extrusion is performed in the presence of an absorbent instead of water) via continuous HME processing has been reported [12]. In this approach, inorganic excipients such as Neusilin® and/or Fujicalin® can be used as novel inorganic carriers [10, 15–17] to produce amorphous drug-containing granules almost in free-flowing ready-to-use nature [11, 12]. In this process, inorganic carrier can also be used to manufacture homogenous one phase amorphous systems when combined with a hydrophilic polymer such as HPMC [11].

Previous studies show that X-ray diffraction has largely been used to quantify the amorous content in various formulations both quantitatively and qualitatively [12, 13]. Similarly, solution calorimetry (SolCal) can be used as an ideal technique to quantify the amorphous content in the granules prepared via TSG which is to the best of our knowledge, has not been done before. SolCal refers to the determination of heat of solution when a solid is dissolved in a liquid, or two liquids are mixed. The software in solution calorimetry technique uses heat balance equations [18] to determine the change in enthalpy from the change in offset temperature as a function of time. Various reports have been published [19–21] undertaking research with solution calorimetry using sucrose, lactose, their physical form especially crystalline or amorphous, their water contents, calibration plot, etc. A study demonstrated heat of solution of carbamazepine (CBZ) and later [22] relative enthalpy of formation of co-crystals of CBZ: SAC (saccharin) elsewhere. Later in 2013, Majumder et al. have carried out extensive work with solution calorimetry and demonstrated heat of solution of different co-crystal systems using different physical forms of the starting materials [23]. This technique is capable of determining the amorphous content in the formulation more accurately and precisely, but somewhat surprisingly, the widespread use of this innovative technology in amorphous granules prepared via continuous TSG has not been explored yet.

Thus, the aim of the current study is to implement SolCal to determine amorphous content of benzoyl-methoxy-methylindol-acetic acid (BMA)—a model poorly soluble BCS class II and highly crystalline drug, in the granules prepared via a continuous twin-screw dry granulations. An attempt has also been made for the first time to interrelate the findings from the SolCal to that of the in vitro release of the drug from the amorphous granules.

## Materials and method

### Materials

Benzoyl-methoxy-methylindol-acetic acid (> 99% purity) (BMA) was purchased from Sigma-Aldrich (Batch# 056K1563, Gillingham, UK), and Neusilin US2 (US2) was kindly donated by Fuji Chemical Industries Co., Ltd. (Japan). All solvents used were of analytical grade and used as received.

### Continuous twin-screw dry granulation (TSG)

All granules were prepared via adopting the method described by Maniruzzaman et al. in a previous report with slight modifications [12]. Briefly, all prepared drug/carrier binary mixtures were processed at 180 °C without the die using a twin-screw (L/D 40) extruder (TwinLab 10, Twin-Tech Extrusion Limited, UK). The temperature profiles, screw speed and the torque were recorded for each processed sample. The screw speed used for extrusion was optimised at 100 rpm with a feed rate of ∼0.5–1 kg h^−1^ that resulted in a short residence time of about ~ 45 s. The TwinLab 10 is contacted to a terminal HMI (Omron), and all processing parameters were controlled through the appropriate software supplied by the manufacturer. US2/BMA ratios were optimised at 4, 2.5 and 1 (*w*/*w*) for TSG operation in order to obtain the final granules. The extrudates were optimised and collected in powder forms so that no additional down streaming processing was required.

### Scanning electron microscopy (SEM) and optical microscopy

The surface morphology of the granules was studied via an SEM. For this purpose, the samples were mounted on an aluminium stage using adhesive carbon tape and placed in a low humidity chamber prior to analysis. Microscopy was performed using a JEOL JMS 820 (Freising, Germany) SEM machine with an operating accelerating voltage of 5 kV. Reverse optical microscopy (JEOL JEM1400-Plus, 120 kV, LaB6) was also utilised to investigate the surface of the granules by applying blue, and red filters to visualise the particles as desired. Transmission and fluorescent lights were used to reveal the absorbance, and excitement capabilities of the particles.

### Particle size analysis

Particle size distribution analysis was conducted using a laser diffraction particle size analyser (Sympatec Ltd., UK) equipped with the HELOS sensor and Windox software and was used with the RODOS (dry) system. Detection of the particles was done using the R3 and R5 lenses, which have a particle size detection range of 0.5–175 and 0.5–875 μm, respectively.

### Solid state analysis

The solid state of the drug in the granules was investigated using a Mettler-Toledo 823e (Greifensee, Switzerland) differential scanning calorimeter (DSC). Approximately, 3-5 mg of samples was placed in sealed aluminium pans without lids prior to heating the samples at 10 °C min^−1^ from 0 to 220 °C under dry nitrogen atmosphere. All samples were reheated at the same heating rate and were run in triplicate. The results shown represent the mean.

X-ray powder diffraction (XRPD) was used to determine the crystalline state of the drug in the granulated formulations using X-ray diffractometer (D5000, Siemens, Germany). Samples were scanned over a range of 2*θ* at voltage of 40 kV and current of 30 mA, with scanning angle ranged from 5° to 40° and scan rate of 0.2° s^−1^.

### FTIR analysis

FTIR analysis was performed on the bulk drug, US2, drug/carrier physical mixtures and the manufactured granules in order to assess any possible drug excipient interactions using a PerkinElmer PE1600 (Massachusetts 02451, USA) Fourier transform infrared spectrometer via obtaining KBr disc method. The disc was prepared from the powders by pressing it for 5 min in a constant pressure with the aid of a hydraulic press. The spectra were acquired from 400 to 3600 wavelength cm^−1^ range. All samples were run triplicate and the results shown represent the mean value.

### Dynamic vapour sorption (DVS) analysis

Approximately 25 mg of sample was placed into a glass balance pan and loaded into a DVS Advantage System (SMS) held at 25 ± 0.1 °C. The sample was then subjected to a step profile change from 0 to 90% RH (P/Po, whereby *P* = partial pressure of water vapour and Po = equilibrium vapour pressure of water) at 10% RH increments, followed by desorption from 90% RH to 0% RH at 10% RH decrements maintaining the sample at drying step at 25 °C. The weight change during the sorption cycle was then monitored, allowing for the hygroscopic nature of the sample to be determined. The % RH was maintained by the mixture of DI water and dry nitrogen (flow rate of 200 sccm) which acted as a wet purge and dry purge, respectively. The percentage of mass change per minute (dm/dt) was set as 0.002 with a minimum stability for 30 min, and maximum stage time was 240 min at each % RH.

### Solution calorimetry

A glass ampoule containing 100 mg ± 1 mg of the sample (accurately weighed) was sealed with a silicon bung and further sealed using molten beeswax. The ampoule was then loaded into the stirrer unit of the solution calorimeter. This unit was then lowered into the glass vessel containing exactly 100 ml of solvent (DMSO in this case) with the glass ampoule isolating the solute from the solvent. The combined unit was then lowered into the experimental chamber for equilibration prior to the experiment, with the stirrer speed set at 500 rpm. The solvent was heated to − 180 mK (offset temperature using the inbuilt heater) by applying heater power of 500 mW. Calibrations of 500 mW/10 s (= 5 J) were applied before and after the break with baselines of 5 min before and after, a break time of 5 min was used, with a baseline of 5 min after. Once an exponential fit over a 5 min period with a standard deviation of less than 10 μK had been attained, the experiment was then conducted and the resulting heat of solution determined. Dissolution of the sample was performed by breaking the ampoule. The heat of solution was reported in joules per gram. The instrument is calibrated quarterly using crystalline and amorphous sucrose in de-ionised water (DI water) for endothermic and exothermic reactions, respectively.

## Results and discussion

### Continuous granulations and the particle morphology

The final optimised processing temperature of the extruded granules was set at 180 °C, as previously, this temperature was found most appropriate in order to process and obtain amorphous granules [12]. The unique formulation compositions and the drug/carrier miscibility produced a completely homogenous system in a free-flowing granulated forms. This could be attributed to the high flowability (Carr’s index above 10) of US2 that contains Al_2_O_3_ (~ 30%) enabling it to adsorb the BMA molecules and thus dry up the whole system and eventually evacuate the extrudates in free-flowing powder forms [12]. It has been reported that the tetrahedron or octahedron-shaped Al_2_O_3_ forms a complex three-dimensional structure in US2 making the compound an excellent proton donor or acceptor [24]. As a result, US2 facilitates a completely dry blending process in extrusion which can add a new insight for pharmaceutical research and development when TSG is involved by eliminating any further down streaming processing steps. Whilst the down streaming processing during a typical HME is considered as one of the major drawbacks, can perhaps, be remarkably absent in foregoing process making this innovative idea to enjoy a resurgence in the future formulation strategy when US2 is used as a novel carrier excipient.

**Fig. 1:**
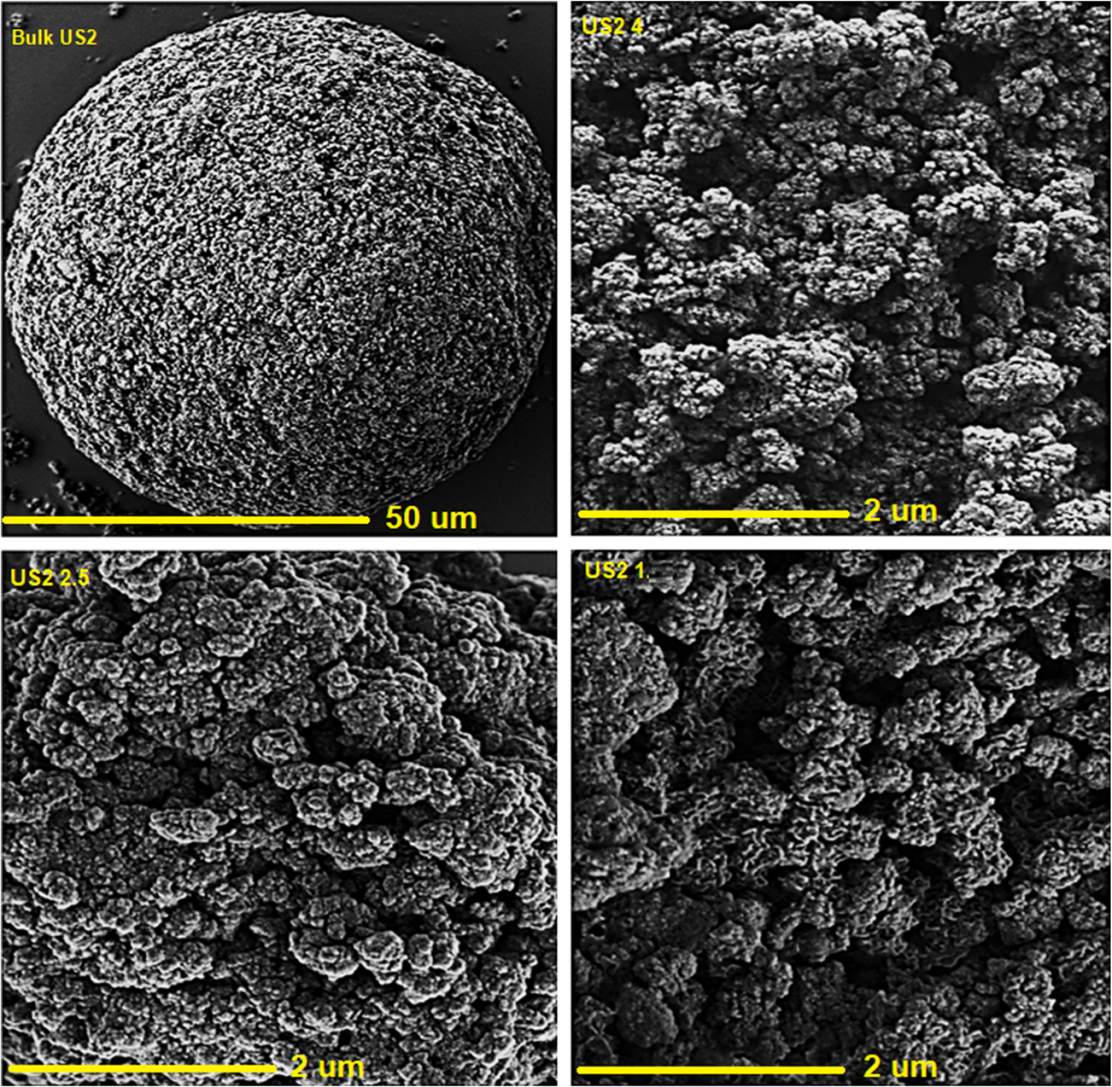
SEM images of US2 bulk and BMA/US2 extruded granules

The surface morphology of the produced granules examined via SEM showed no drug crystals on the extrudate surface. Rather, it showed agglomerated granules in irregular shapes. There was also no evidence of the different phases in any of the granules which could be attributed to the intensive high shear mixing during the processing. Also, this could be related to the drug entrapment into the meso-porous US2 network with high specific surface area (300 m^2^ g^−1^) during the processing. Interestingly, at a very high resolution, the particle size has dramatically fallen into nanoscale (Fig. 1) and the granules are more agglomerates of nano-sized particles. This foregoing phenomenon can eventually be of great interests in oral drug delivery systems and thus opening a new scope of utilising nano-technology via TSG approach.

**Fig. 2:**
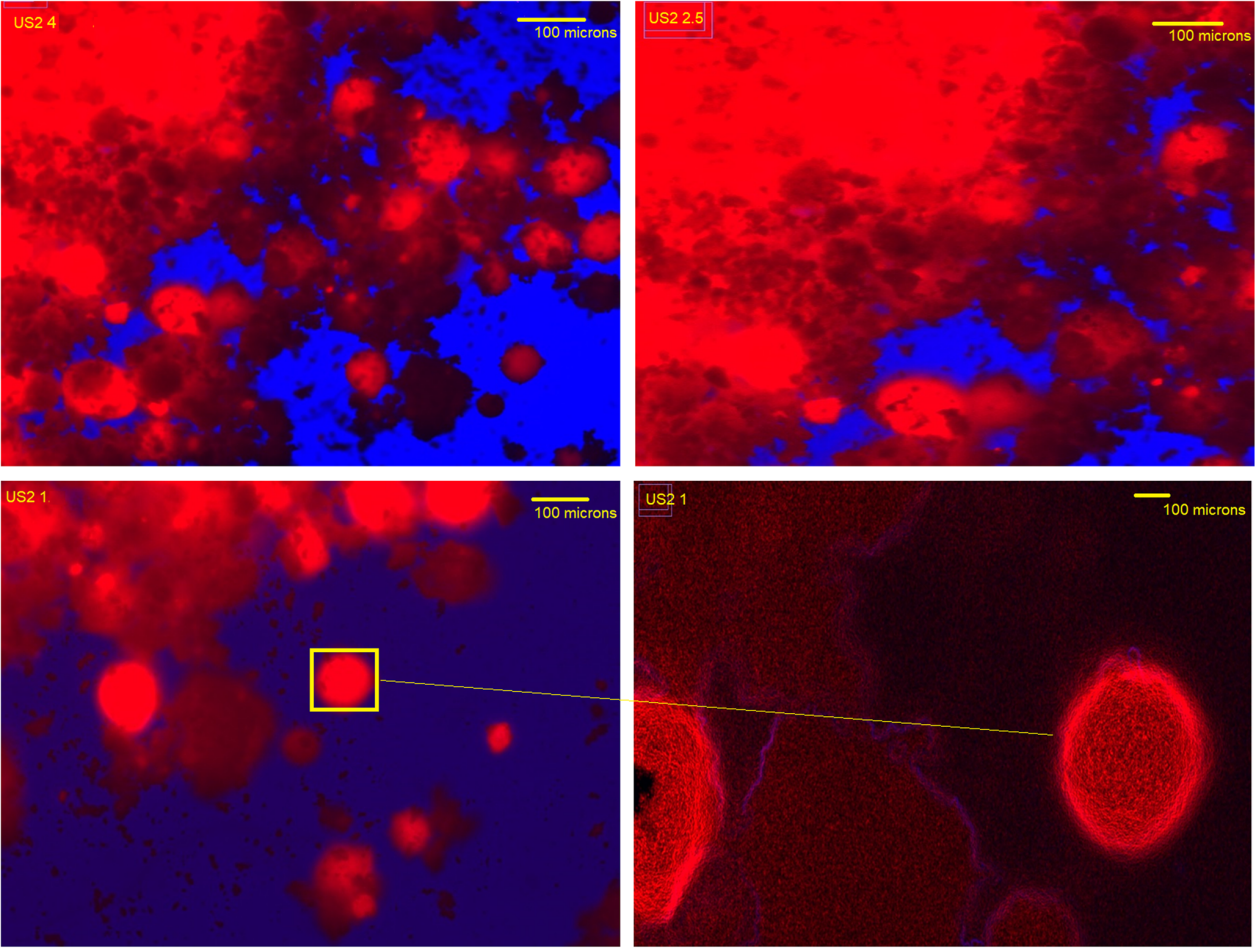
Fluorescence microscopic images of BMA/US2 granules produced via HME

Reverse optical microscopy examined the surface morphology of the granulated particles. As can be seen in Fig. 2, the fluorescent particles of US2 present in the formulations are spherical and porous. There are some particles adsorbed on the surface of fluorescent spherical particles which could be the drug particles. These images also show the homogeneous distribution and uniformity of drugs within the carrier matrices in all of the formulations. In general, at a molecular level, absorbance relates to energy status of the particles and their ability to excite the electrons within their orbital. As a result, it is seen that the US2 particles are porous, and BMA molecules may have been adsorbed or entrapped within the porous structure of US2.

**Fig. 3:**
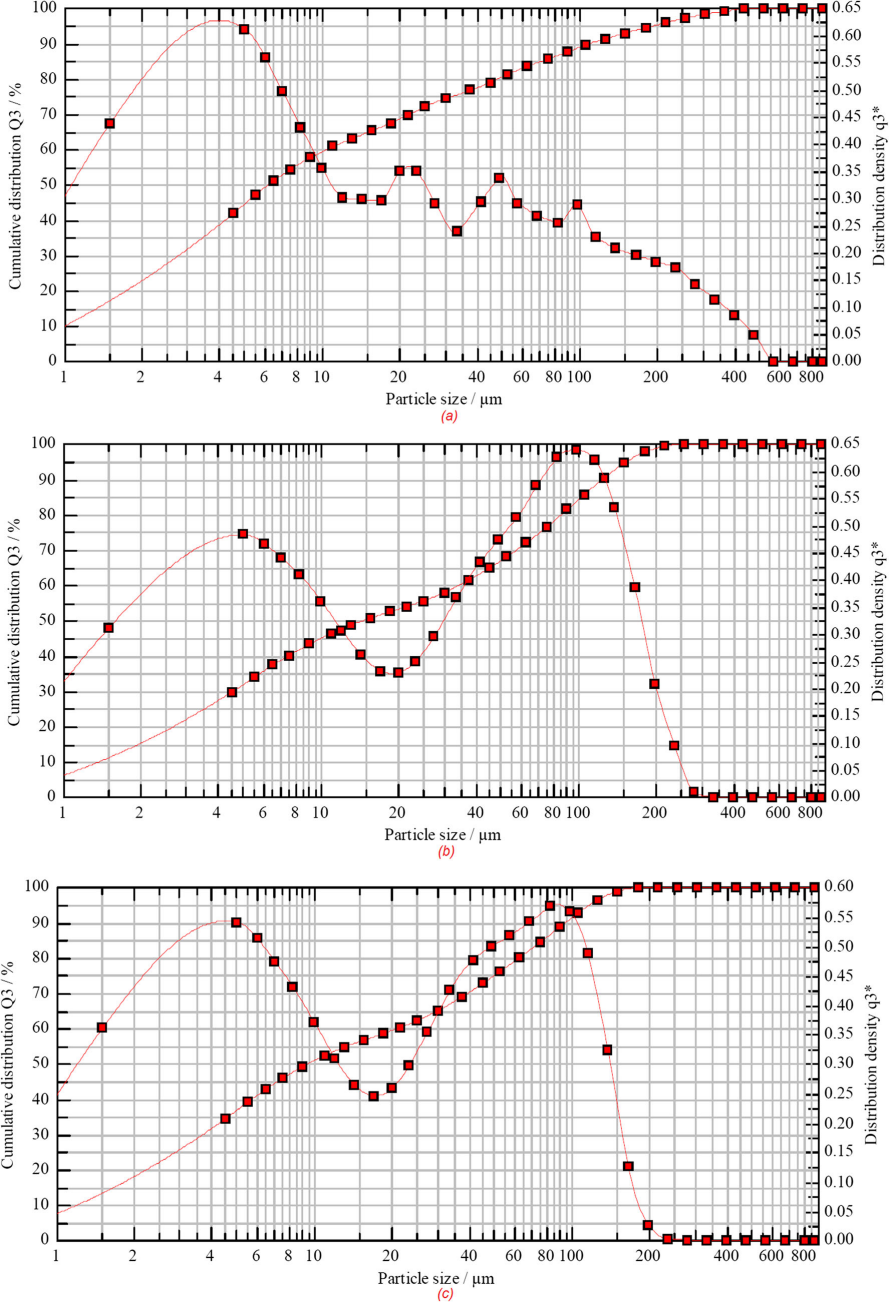
Particle size distribution of extruded formulations processed via dry granulations processing. **a** US2 4. **b** US2 2.5. **c** US2 1

The particle size distribution and *d*_10_, *d*_50_ and *d*_90_ values were determined for all extruded granules. As depicted in Fig. 3, data obtained from the analysis showed the particle sizes lower than 115 μm for all formulations which is quite similar to that of US2 (*d*_90_ 130 μm). In contrast, the bulk BMA showed very fine particles with a narrow mono-modal distribution and *d*_90_ 22 μm. A small percentage can be seen at sizes > 300 μm due to some agglomerates produced during the granulation process. Nevertheless, all extruded granules were within the range suitable for the oral solid dosage forms and showed excellent flow properties.

### Solid state analysis

**Fig. 4:**
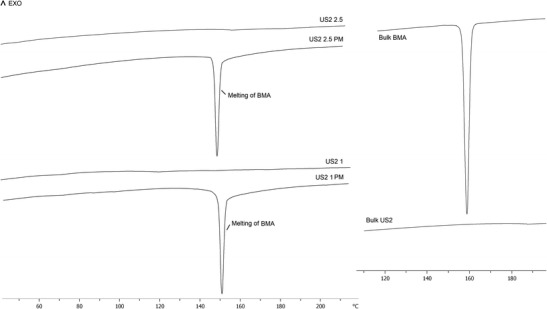
DSC transitions of bulk BMA, US2, physical blends of the formulations and extruded granules

The solid state of the bulk compounds, physical mixtures (PM) and the extruded granulated formations were examined via DSC. The DSC thermograms of bulk BMA showed sharp melting transitions at ~ 162.1 °C with a fusion enthalpy (Δ*H*) of about ~ 101.1 J g^−1^ (Fig. 4). The analysis of the combined heat flow of the bulk MAS showed no crystalline melting endotherms indicating its amorphous nature [25, 26]. All drug–carrier physical mixtures exhibited sharp melting transitions at slightly shifted towards lower temperature in the range of 150–155 °C corresponding to the melting of the crystalline BMA. The heat of fusion increased with the increase of the drug content in the formulations. For example, US2 4 (20% BMA) showed an enthalpy (Δ*H*) of ~ 11 J g^−1^, whereas US2 1 (50% drug) showed Δ*H* of about ~ 29 J g^−1^ which is in line with the theoretical calculations. In contrast, there were no endotherms observed in any of the extruded granules. The absence of melting transitions corresponding to BMA in all of the formulations indicates the absence of the crystalline BMA and thus the development of amorphous system [26]. Moreover, the absence of multiple Tgs in the formulations indicates that the drug is entrapped within the porous network of the inorganic carrier US2, as the Tg of the US2 is well above the temperature range used in this study (~ > 300 °C). Nonetheless, it can be claimed that the US2 can successfully be used as a novel inorganic carrier to manufacture amorphous drug-containing granulated formulations.

**Fig. 5:**
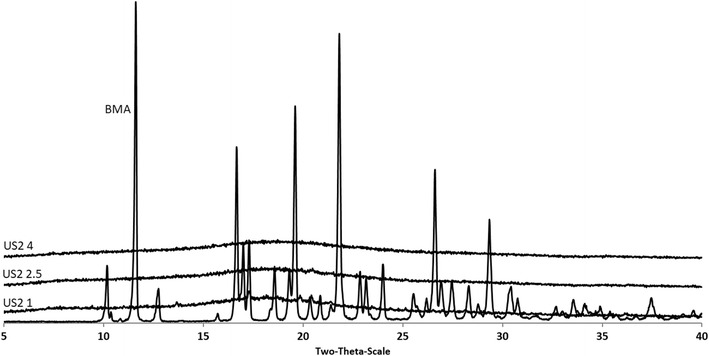
XRPD diffractograms of bulk BMA, US2 and all BMA/US2 formulations

Furthermore, the US2-based extruded granules, bulk drug and the corresponding physical mixtures of the same compositions were studied by X-ray analysis in order to examine the crystallinity of the drug in the formulations. All diffractograms were recorded to examine BMA crystalline state. As can be seen in Fig. 5, the diffractogram of bulk BMA showed distinct characteristic intense peaks at various 2*θ* positions indicating that the BMA is highly crystalline. Similarly, the physical mixtures of all BMA formulations showed identical but low intense peaks suggesting that the drug retains its crystallinity in the physical blends. In contrast, no distinct intensity peaks were observed in the diffractograms of the extruded formulations even at high drug loadings e.g. in US2 1 with 50% *w*/*w* drug. The absence of BMA intensity peaks indicates the presence of its amorphous state or molecularly dispersed state into the US2 matrices after the granulation process was optimised. The results obtained in the XRPD complement the findings from DSC analysis.

### FTIR analysis

**Fig. 6:**
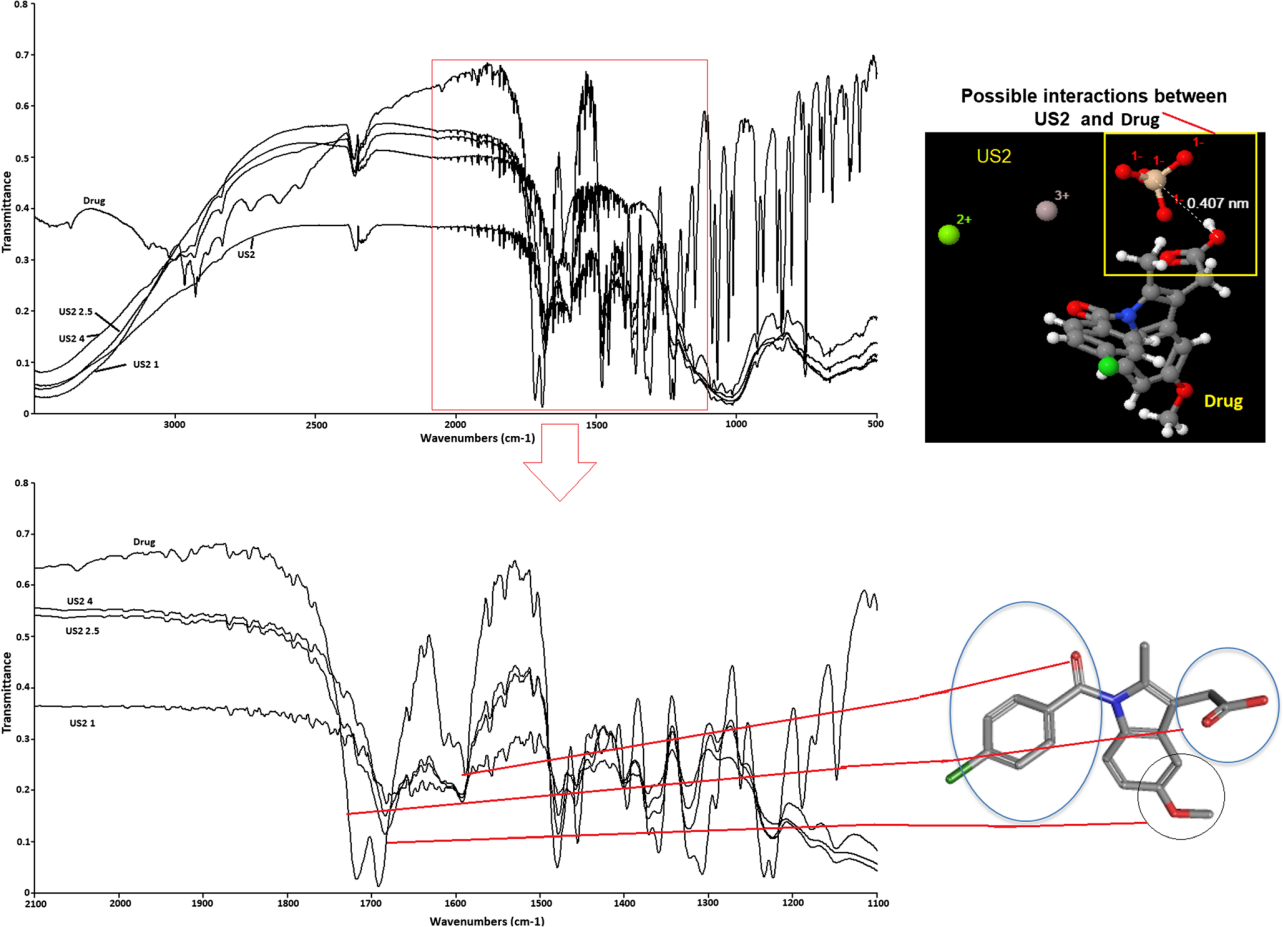
**a** FTIR spectra of bulk BMA, US2 and all extruded formulations. **b** Molecular modelling of the drug and excipient to outline the possible interactions

The drug BMA contains both phenyl carbonyl and carboxylic acid groups in the structure (Fig. 6) [12, 26]. The FTIR spectra of the crystalline BMA showed the acid dimer peak at 1720 and at 1690 cm^−1^, respectively (Fig. 6). In addition, the peak at 1585 cm^−1^ was attributed to the benzoyl carbonyl group attached to a nitrogen atom. The absorbance of the drug dimer peaks disappeared after the granulation process was optimised in all formulations, and only one slightly shifted peak at 1680 cm^−1^ was visible. This could be attributed to the intermolecular interactions associated with crystalline BMA. Interestingly, the increase in the concentration of the drug in formulations showed an increase in the intensity of the characteristic peak at 1680 cm^−1^ which could be due to the presence of more NH– in the formulation as it only is present in the BMA. Previous study revealed that owing to the close proximity of the pKa values of SiO_2_ to that of BMA, the silanol group from US2 can become amphoteric, functioning as a Bronsted acid or as a Bronsted base [12, 27]. Therefore, due to the local electronegativities of –COOH and –Si-OH from the drug and US2, respectively –C-O-Si is formed as a result of ionic bonding. These foregoing claims have been outlined in the molecular modelling predictions as depicted in Fig. 6. As can be seen in the image, depending on the initial positioning of the BMA molecule, multiple bonding patterns were identified. However, after energy optimisation, the highest proximity has been observed for the interaction between the silanol group of the carrier and the carboxyl group of the drug (~ 4.07 Å). It has also been outlined that both the carbonyl group and amine group within the drug molecule could form bonds (e.g. non-covalent H bonds) with the –Si-OH group in the BMA, as indicated by the optimal distances between the bond donor and acceptor ~ 15.08 and ~ 11.50 Å, respectively.

Also, the benzoyl carbonyl peak at 1585 cm^−1^ becomes broader as a function of decreasing drug concentrations, indicating increasing amorphicity of BMA. It can be claimed that the higher the BMA amount, the sharper the benzoyl carbonyl peak thus stronger the interactions. The high intense stretch at 1680 cm^−1^ in US2 1 where the drug content is the highest (50% *w*/*w*) indicates that intermolecular interactions are favoured with the increase in the BMA concentration. In contrast, as expected, the characteristic dimer peaks of BMA are present at the same position for all PM formulations (data not shown).

### DVS analysis

**Fig. 7:**
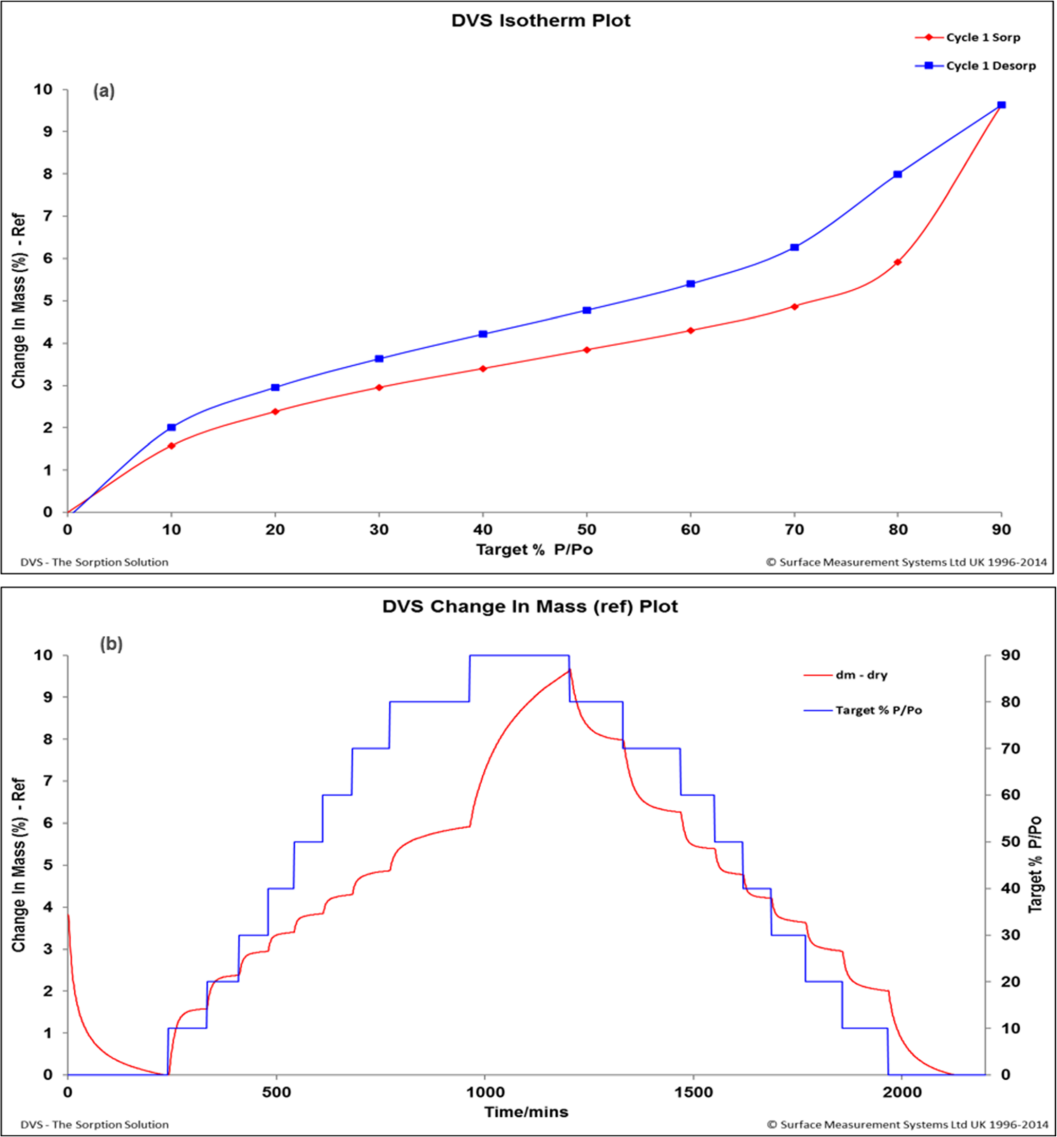
**a** Water sorption isotherms of formulation US2 4. **b** Water sorption kinetics of formulation US2 4

The DVS data presented in Fig. 7a, b showed that the US2 2.5 BMA (~ 30% *w*/*w*) formulation sample desorbed ca. 4% *w*/*w* moisture at 0% RH suggesting that the sample already contained this moisture. This is confirmed by the water uptake phenomenon, ca. 4% *w*/*w* up to 50% RH, which implies that at ambient condition, this sample is hygroscopic. The hygroscopic nature is also confirmed by the hygroscopicity classification as per the Ph. Eur. in Table 1 [28]. So, a care must be taken to formulate and store this sample after production during future developmental work. A further water uptake up to 6% *w*/*w* at 80% RH, followed by a sharp increase in weight gain up to ca. 10% *w*/*w*, which even did not reach equilibrium, demonstrates that this sample has an affinity to absorb large quantity of moisture upon exposure to high humidity. Interestingly, no weight loss was observed to suggest crystallisation of an amorphous material, due to water acting as a plasticiser, indicating that water is not a good plasticiser for this material. Importantly, this implies that this formulation could be a stable one over a long period of time at high humidity if water absorption is controlled. The sample did not hold onto any gained mass during desorption cycle suggesting that the sample is possibly non-porous in nature and post-DVS sample also did not show any obvious physical appearance change. Similar results were obtained for other formulations. The effect of the moisture on the solid state of the extruded granules showed that the manufactured formulations are stable even at higher drug concentration (up to 50% *w*/*w*).

**Table 1:** Hygroscopicity classification

Classification	Weight increase at 80% RH (25 °C)
Non-hygroscopic	< 0.2%
Slightly hygroscopic	≥ 0.2 and < 2%
Hygroscopic	≥ 2 and < 15%
Very hygroscopic	≥ 15%
Deliquescent	Sufficient water is absorbed to form a liquid

From [28]

### Solution calorimetry (SolCal)

The solution calorimetry data presented in Table 2 suggested a reproducible data set for a biphasic system. It clearly demonstrates and distinguishes heat of solution of different materials. There are two interesting aspects of these data set: the first one being all formulation samples with different % *w*/*w* of the API loading showed a response which is different to physical mix (theoretically expected), and had there been a physical mix made for those formulation samples as their theoretical response were different than the experimental response sugggesting that the formulations were indeed not a mere physical mix. This hypothesis is effectively validated by the data obtained for 50:50 (*w*/*w*) physical mix (PM) between BMA and US2. The second aspect is that the US2 1 with 50% *w*/*w* drug did not follow the expected trend as the other US2 4 and US2 2.5 samples suggesting that the API load was optimised up to ~ 30% *w*/*w* or in between 30 and 40% *w*/*w*.

**Table 2:** Solution calorimetric data of US2 samples

Sample	Trial	Weight (g)	Heat of solution (∆*H*) in J g^−1^ obtained experimentally						Theoretical heat of solution (∆*H*)
			Pre-break calibration	Post-break calibration	Mean	Overall mean	SD	RSD	
BMA	1	0.10026	36.005	35.998	36.00	36.6	0.5	1.4	N/A
	2	0.10034	36.947	36.974	36.96				
	3	0.10036	36.888	36.839	36.86				
US2	1	0.10062	− 45.948	− 45.874	− 45.91	− 46.5	0.5	− 1.1	
	2	0.10064	− 46.869	− 46.847	− 46.86				
	3	0.10053	− 46.670	− 46.656	− 46.66				
US2 1 (PM)	1	0.10013	− 5.951	− 5.936	− 5.94	− 5.4	0.5	− 9.0	− 4.9
	2	0.10186	− 4.974	− 4.969	− 4.97				
	3	0.10071	− 5.369	− 5.356	− 5.36				
US2 4	1	0.10072	− 10.823	− 10.809	− 10.82	− 10.9	0.3	− 2.8	− 29.9
	2	0.10070	− 10.726	− 10.702	− 10.71				
	3	0.10050	− 11.293	− 11.277	− 11.29				
US2 2.5	1	0.10008	− 15.983	− 15.968	− 15.98	− 16.3	0.6	− 3.9	− 21.6
	2	0.10035	− 17.014	− 16.986	− 17.00				
	3	0.10009	− 15.866	− 15.850	− 15.86				
US2 1	1	0.10045	− 14.052	− 14.003	− 14.03	− 14.4	0.4	− 3.1	− 13.2
	2	0.10045	− 14.911	− 14.894	− 14.90				
	3	0.10092	− 14.292	− 14.276	− 14.28				

**Fig. 8:**
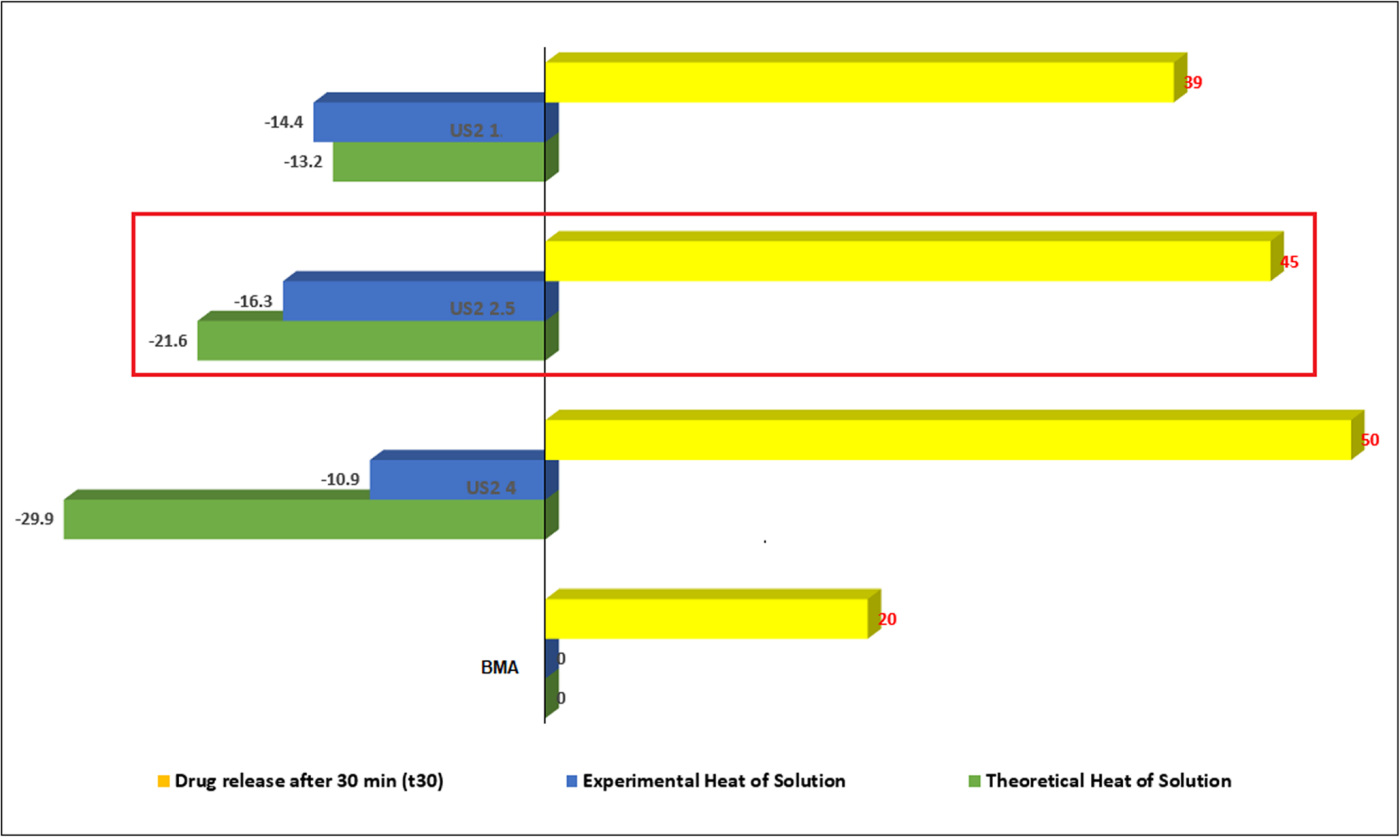
Heat of solution data of all formulations and interrelation with the drug release at *t*_30_ (*n* = 3)

Generally, amorphous samples exhibit exothermic response; it is possible that the US2 2.5 sample is higher in amorphous nature than its US2 4 and US2 1 counterparts. However, the reason could be different in both cases. Based on these data, the relationship between 20% *w*/*w* (US2 4) and 30% *w*/*w* (US2 2.5) loaded samples is that US2 2.5 sample is higher in amorphous than the US2 4 and, therefore, will be stable for longer as a solid dispersion product. This foregoing claim complements the finding from the release data of BMA achieved at 30 min. As can be seen in Fig. 8, the release of the drug was increased up to the 30% *w*/*w* drug loading (US2 2.5). With the increase in the content of the drug, the release seemed to have decreased possibly due to the drug collapsing into its crystalline form. However, anything above 40% *w*/*w* drug loaded samples showed that the optimum level of drug loading has been achieved and the sample will no longer exhibit higher amorphous nature or exothermic response as compared to 30% *w*/*w* loaded sample. These data suggest that the solution calorimetry could be effectively used to distinguish different solid dispersion products with varying drug loadings and be interrelated with the most optimised formulation as a function of the increased release of the drug. As the name suggests, it is a precision solution calorimetry technique; therefore, room temperature, method development, sample preparation, solvent selection, etc. require years of experience to reproduce consistent data especially in complex systems like solid dispersion, granulated formulations using this technique.

## Conclusion

In the current study, US2 was demonstrated as a suitable alternative carrier for the development of the amorphous granules by means of continuous dry granulation approach. The drug has been found to exist in its amorphous forms as determined by DSC, XRPD where as both SEM and optical microscopic analyses revealed a homogenous dispersion of the drug into the carrier matrices due to the intense mixing during the granulation processing. The analysis conducted via the FTIR outlines a possible intermolecular interaction between the drug and the carrier which may have been the underlying reason of the development of the stable solid dispersions. The results obtained from the DVS suggest that the formulations are quite stable even at a relatively high drug loading (up to 50% *w*/*w*). Moreover, the results obtained from the novel SolCal analysis revealed and interrelated the amorphous contents to that of the dissolution of the drug from the formulations very precisely. It is concluded that SolCal can be used as a powerful tool to determine the amorphous content as a function of the heat of solution in order to develop stable amorphous solid dispersions with enhanced dissolution rates.
